# Possible mechanism of static progressive stretching combined with extracorporeal shock wave therapy in reducing knee joint contracture in rats based on MAPK/ERK pathway

**DOI:** 10.17305/bjbms.2022.8152

**Published:** 2023-03-16

**Authors:** Run Zhang, Quan-Bing Zhang, Yun Zhou, Rui Zhang, Feng Wang

**Affiliations:** 1 Department of Rehabilitation Medicine, The Second Hospital of Anhui Medical University, Hefei, China; 2 Research Center for Translational Medicine, The Second Hospital of Anhui Medical University, Hefei, China; 3 School of Basic Medical Sciences, Anhui Medical University, Hefei, China

**Keywords:** Joint contracture, joint capsule, static progressive stretching (SPS), extracorporeal shock wave therapy (ESWT), mitogen-activated protein kinase (MAPK)/extracellular signal-regulated kinase (ERK) pathway

## Abstract

The study aimed to observe the therapeutic effect of static progressive stretching (SPS) combined with extracorporeal shock wave therapy (ESWT) on extension knee joint contracture in rats and the effect on the mitogen-activated protein kinase (MAPK)/extracellular signal-regulated kinase (ERK) pathway in the development of joint capsule fibrosis. Thirty-six Sprague Dawley rats were randomly divided into blank control group, immobilization model group, natural recovery group, ESWT intervention group, SPS intervention group, and SPS combined with ESWT intervention group. The left knee joints of the rats, except for the control group, were fixed with an external fixation brace for four weeks at full extension to form joint contracture. The therapeutic effect of each intervention was assessed by evaluating total and arthrogenic contracture, the number of total cells and collagen deposition in the anterior joint capsule, the protein levels of TGF-β1, FGF-2, and ERK2 in the anterior joint capsule, the mean optical density of upstream RAS and downstream ERK2 positive expression in the MAPK/ERK pathway. SPS in combination with ESWT was more effective in relieving joint contracture, improving the histopathological changes in the anterior joint capsule, and suppressing the high expression of target proteins and the overactivated MAPK/ERK pathway. The overactivated MAPK/ERK pathway was involved in the formation of extension knee joint contracture in rats. SPS in combination with ESWT was effective in relieving joint contracture and fibrosis of joint capsule. Moreover, the inhibition of the overactivated MAPK/ERK pathway may be the potential molecular mechanism for its therapeutic effect.

## Introduction

Joint contracture is a fibrotic disease characterized by limitation in range of motion (ROM) of the joint [1, 2]. Once developed, the patients’ quality of life is greatly affected, permanent contracture with continuous deterioration develops, and the patient becomes permanently disabled [1, 3]. Among many causes of joint contracture, joint immobilization is the most common one [1–4]. In the process of joint immobilization, arthrogenic and myogenic components are involved in the occurrence and development of joint contracture [1, 3]. The myogenic components mainly limit the ROM in short-term immobilization, which can return to the normal level after remobilization; the arthrogenic components play a dominant role in long-term immobilization, which cannot be effectively relieved even with a prolonged remobilization [5]. Compared with the myogenic components, the arthrogenic components are more difficult to recover, which are the key points and difficulties in the treatment of joint contractures [6]. Among the arthrogenic components, joint capsule fibrosis is unanimously considered as the decisive factor [7]. The exact pathophysiological mechanism and new treatment methods for joint contracture are the goal on which doctors and therapists need to work.

The mitogen-activated protein kinase (MAPK)/extracellular signal-regulated kinase (ERK) pathway is a major pathway in the control and regulation of cellular processes associated with fibrosis in some fibrotic diseases. The overactivated MAPK/ERK pathway can promote the occurrence and development of fibrosis [8–10]. Transforming growth factor beta-1 (TGF-β1) is a recognized profibrotic cytokine [1, 8, 9], which can stimulate the collagen expression of fibroblasts through the Smad pathway, and can cooperate with fibroblast growth factor 2 (FGF-2) to promote the proliferation of fibroblasts through the MAPK/ERK pathway [11]. Sun et al. [12] treated the rat model of post-traumatic knee joint contracture by ERK2 siRNA intervention, which can effectively reduce the joint capsule fibrosis. We hypothesized that the overactivated MAPK/ERK pathway plays a key role in the formation of joint capsule fibrosis.

Extracorporeal shock wave therapy (ESWT) is widely used in musculoskeletal system diseases [13, 14]. The mechanical stimulation produced by ESWT has biochemical effects on some cells and affects the cell cycle and metabolic pathways, which can regulate the migration, proliferation, differentiation and apoptosis of fibroblasts, endothelial cells, osteoblasts, and tenocytes [15]. Relevant studies have shown that ESWT can reduce cell migration and effectively inhibit the expression of TGF-β1, alpha smooth muscle actin (α-SMA), type I collagen, and fibronectin [16], making it a feasible and cost-effective method for the treatment of pathological scars after burns [17]. In addition, ESWT can significantly and effectively reduce the fibrosis in the treatment of epidural fibrosis [18] and capsular fibrosis after insertion of silicone implants [19]. Huang et al. [13] found that radial ESWT combined with ultrashort wave therapy could inhibit the high expression of TGF-β1 and hypoxia-inducible factor 1 alpha (HIF-1α), and radial ESWT could inhibit the NF-κB/HIF-1α signaling pathway [14], which could reduce the myogenic contracture after long-term immobilization. A study reported that the adhesion between the lateral femoral condyle and the joint capsule was significantly lower than that of the control group after ESWT during the repair of the knee in rabbits [20]. It is reasonable to believe that ESWT has positive effect on arthrogenic contracture.

**Figure 1.**
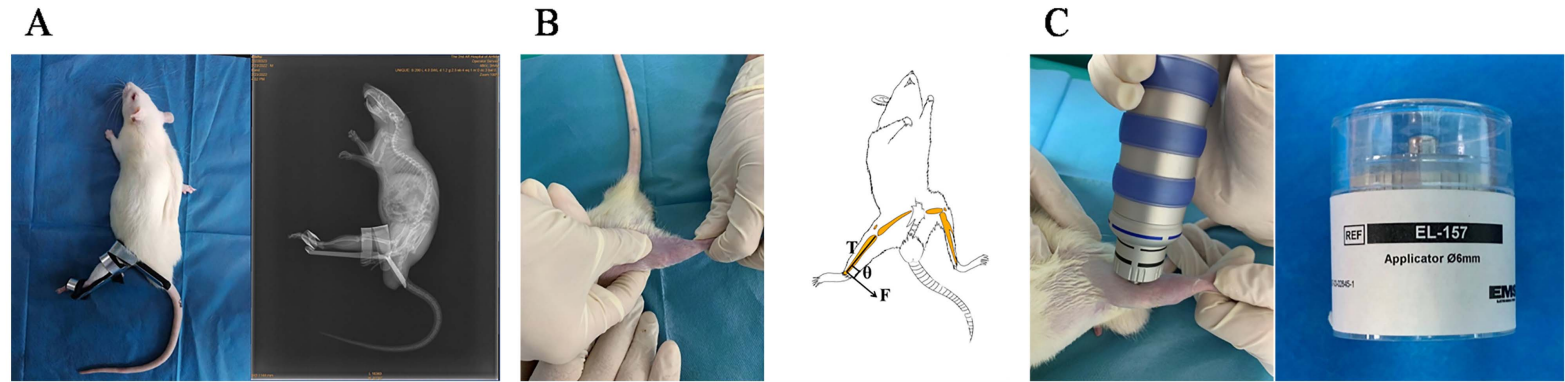
**Schematic diagram of knee joint contracture model and interventions**. (A) The rat model of the extension knee joint contracture and observation under X-ray film; (B) Static progressive stretching intervention after the removal of the external fixation brace of the left knee joint of the rat; T is the long axis of the tibia, F is the stretching force, *θ* ═ 90∘; (C) Extracorporeal shock wave intervention after the removal of the external fixation brace of the left knee joint of the rat and the type of the treatment probe.

It is well known that stretch therapy is the main way to improve the ROM of joint in joint contracture [21], and can also effectively relieve hyperalgesia and muscle damage caused by joint immobilization [22]. In addition, stretching can effectively prevent the shortening and adhesion of the joint capsule and reduce joint capsule fibrosis [23]. Static progressive stretching (SPS) uses the principle of stress relaxation, which improves the ROM within a limited treatment time more effectively [24], and it also has significant therapeutic effect in relieving pain and stiffness [25]. Numerous clinical studies have demonstrated the effectiveness and superiority of the SPS therapy [24–26]. At present, there are few reports on basic research of SPS and the application of its unique stress relaxation principles in treatment.

This study combined the biochemical effects of ESWT on specific cells and the effectiveness and superiority of SPS, to explore whether it can improve the joint contracture and joint capsule fibrosis by inhibiting the overactivated MAPK/ERK pathway. The study explored the immobilization-induced extension knee joint contracture model in rat from the following aspects: (1) improvement of total contracture and arthrogenic contracture; (2) improvement of the histopathological features in the anterior joint capsule tissue; (3) changes in protein levels of TGF-β1, FGF-2, and ERK2 in the anterior joint capsule tissue; (4) changes in the mean optical density (MOD) of upstream RAS and downstream ERK2 protein in the MAPK/ERK pathway.

## Materials and methods

### Experimental design

A total of 36 skeletal mature male Sprague Dawley rats (Experimental Animal Center of Anhui Medical University, Hefei, China, 12 weeks old, weight 250–300 g) were used in this experiment. Rats were housed in standard cages (room temperature 24 ^∘^C–25 ^∘^C, 12 h light/dark cycle) and had free access to water and food. Thirty randomly selected rats were anesthetized by intraperitoneal injection of 300 mg/kg 10% chloral hydrate and then their left knee joints were fixed for 4 weeks at full extension by the fixation brace (Patent No.202120470158.0) and fixation method was noted in the previous studies [27] (Figure 1A). The health status of the rats and the immobilization of the brace were observed daily, and the immobilization strength was adjusted in time to ensure effective immobilization. All rats were in good health, and none died. After 4 weeks, the external fixation brace was removed, and 30 fixed rats were randomly assigned to the immobilization model group (IM), the natural recovery group (NR), the ESWT intervention group (EI), the SPS intervention group (SI), and SPS combined with ESWT intervention group (CI), and 6 rats without left knee joint fixation were assigned to the control group (C). The C group was euthanized after eight weeks of free movement in the cage. The IM group was euthanized immediately after removing the external fixation brace. The NR group was euthanized after four weeks of free movement in the cage after removing the external fixation brace. The EI, SI, and CI groups received ESWT intervention, SPS intervention, and SPS in combination with ESWT intervention for four weeks, respectively (Table 1), and all rats were euthanized after four weeks of intervention. Euthanasia was performed by intraperitoneal injection of 400 mg/kg 10% chloral hydrate [27].

**Table 1:** Intervention methods and sample size in each experimental group

**Grouping**	**Quantity**	**Joint Immobilization**	**ESWT**	**SPS**
Group C	6	−	−	−
Group IM	6	+	−	−
Group NR	6	+	−	−
Group EI	6	+	+	−
Group SI	6	+	−	+
Group CI	6	+	+	+

“+” means yes and “−” means no. C: Control group; IM: Immobilization model group; NR: Natural recovery group; EI: Extracorporeal shock wave intervention group; SI: Static progressive stretching intervention group; CI: Static progressive stretching combined with extracorporeal shockwave intervention group.

**Figure 2.**
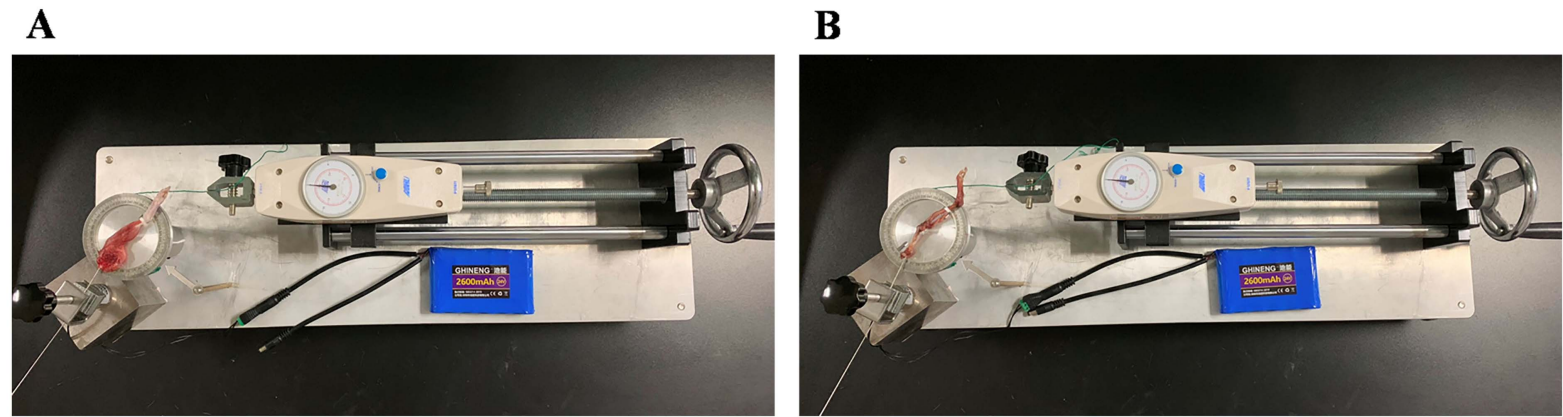
**Measurement of range of motion.** (A) Measurement of range of motion of the left knee joint before myotomy; (B) Measurement of range of motion of the left knee joint after myotomy.

### Experimental interventions

#### Static progressive stretching

A total of 12 rats in the SI and CI groups were treated with SPS 5 times a week for 4 weeks. Before the SPS intervention, the rats were placed and fixed on the treatment table, and the SPS intervention was performed by a therapist with 9 years of professional experience. The thigh was immobilized with one hand and the distal end of the calf was pinched with the other, and a force was applied perpendicular to the long axis of the tibia to stretch the knee in flexion (Figure 1B). The stretching intensity was adjusted according to the tension of the soft tissues around the knee joint and the reaction of the rats. The flexion angle was adjusted after five minutes of static stretching to further increase the angle, followed by another five minutes of stretching, and so on until the angle stopped increasing. The process was repeated and the total duration of the treatment was 30 min.

#### Extracorporeal shock wave therapy

A total of 12 rats in the EI and CI groups were treated with ESWT twice a week for 4 weeks. A pneumatic ballistic shock wave device (Swiss Dolor Clast Smart, Switzerland) was used in the experiment. First, the hair around the left knee joint of the rats was cleaned before the treatment, and the skin was not damaged. Second, ultrasound gel was evenly applied to the skin around the left knee joint of the rats, and the diameter of the extracorporeal shock wave warhead was set at 6 mm. After all preparations were completed, ESWT was performed with a pulse dose of 2.5 bar, 6 Hz, 2000 times [13, 14], which is a common therapeutic dose in clinical practice (Figure 1C).

### Measurement of range of motion

Mechanical measuring device (Patent No.ZL202120996643.1) and measuring method for joint ROM, designed in the previous studies, were used in this study [27]. The ROM of the left knee joint in each group was measured before and after myotomy with a standard torque of 0.053 N.m (Figure 2). The following formulas for calculating the degree of total and arthrogenic contracture were used:

Total contracture ═ ROM before myotomy (of the control knee) − ROM before myotomy (of the contracted knee)

Arthrogenic contracture ═ ROM after myotomy (of the control knee) − ROM after myotomy (of the contracted knee)

### Histological analysis

#### Tissue preparation

After measuring the ROM of the left knee joint in each group, the anterior joint capsule of the left knee joint was taken and divided into equal-sized samples. A portion of the samples was fixed with 4% paraformaldehyde overnight at 4 ^∘^C for histopathological examination. The remaining samples were snap frozen in liquid nitrogen and stored at −80 ^∘^C for subsequent molecular protein detection.

#### H&E and Masson staining

Specimens were dehydrated in graded alcohol and embedded in paraffin. Each specimen was cut into a series of 6 µm sections with a microtome, deparaffinized, and then stained with H&E and Masson to detect pathological changes in the total number of cells and collagen deposition of the joint capsule. Sections of each sample were stained following the steps in the instructions for use of the H&E and Masson staining kits and observed under a microscope at 400× magnification (BX43F; OLYMPUS, Tokyo 163-0914, Japan), with 6 fields of view randomly selected and photographed. The number of total cells was represented as cells per square millimeter of joint capsule area, and the collagen deposition was represented as a percentage of the blue area, and analyzed by Image Pro Plus 6.0 software.

#### Immunohistochemical analysis of RAS and ERK2

The treated slice specimens were subjected to high-pressure antigen retrieval in 2000 mL citrate retrieval solution with pH of 6.0, and then incubated in 3% H_2_O_2_ at room temperature to block endogenous peroxidase. Sections were washed 3 times with distilled water and PBS-T, and then incubated overnight at 4 ^∘^C with rabbit anti-ERK2 antibody (1:100; Affinity Biosciences) and rabbit anti-RAS antibody (1:100; Affinity Biosciences), respectively. The sections were washed 3 times with PBS-T and incubated with goat anti-rabbit IgG-HRP (1:200; Affinity Biosciences) as the secondary antibody for 30 min at 37 ^∘^C. After the sections were washed 3 times with PBS-T, DAB chromogenic reagent was added dropwise, and chromogenic time was controlled under the microscope, and the positive result was taken as the degree. Afterward, the sections were stained with hematoxylin for 2–5 min, differentiated in ethanol with 1% hydrochloric acid for several seconds, blued with lithium carbonate solution for 30 s, washed with water, and then dehydrated, hyalinized with xylene, and sealed with neutral gum. Each section was observed under a microscope at 400× magnification (BX43F; OLYMPUS, Tokyo 163-0914, Japan), and 5 fields of view were randomly selected and photographed. Finally, Image-Pro Plus 6.0 software was used to calculate the Sum(IOD) and Sum(Area) values of each picture, and the MOD of RAS and ERK2 were calculated with MOD ═ Sum(IOD)/Sum(Area), which was used as an indicator of their positive expression.

### Protein extraction and western blot detection

The expression of protein levels of TGF-β1, FGF-2, and ERK2 were analyzed by western blot. The samples were grounded into powder with a liquid nitrogen grinder, homogenized in RIPA lysis buffer (WanLeibio, China), centrifuged, and the supernatant collected. Protein quantification was performed with BCA protein assay kit (WanLeibio, China). Next, equivalent amount of protein of each pair of samples was separated by SDS-PAGE on 10% polyacrylamide gels and electrotransferred to polyvinylidene fluoride microporous membranes, then dissolved in TBST after being blocked in 5% skim milk and incubated at room temperature for 2 h. The membranes were incubated with rabbit anti-TGF-β1 antibody (1:2000; WanLeibio), rabbit anti-FGF-2 antibody (1:1000; WanLeibio), rabbit anti-ERK2 antibody (1:1000; WanLeibio), and rabbit anti-GAPDH antibody (1:1000; WanLeibio) at 4 ^∘^C overnight. On the second day, after being washed in TBST solution 3 times for 10 min each time, the membranes were incubated with peroxidase-conjugated affinity-purification goat anti-rabbit IgG-HRP (1:6000; WanLeibio) as the secondary antibody for 45 min at room temperature, and then the membranes were washed three times with TBST solution for 10 min each time. Finally, ECL chemiluminescent droplets were added to the target bands and the signal was detected with digital imaging equipment. The densities of each band were quantified by Image-Pro Plus 6.0 software, and the protein expression levels of TGF-β1, FGF-2, and ERK2 were calculated by comparison with the amount of glyceraldehyde-3-phosphate dehydrogenase (GAPDH) as a loading control.

### Ethical statement

All procedures performed in this animal experiment were approved by the Institutional Animal Care and Use Committee of Anhui Medical University (LLSC20221126).

### Statistical analysis

All experimental data were expressed as mean ± standard deviation, and the data was analyzed and processed with SPSS 26.0 system. All data were in line with normal distribution and homogeneity of variance, which were tested by Shapiro–Wilk method and Levene method. Differences between groups were analyzed by post-hoc SNK test and one-way ANOVA. The total and arthrogenic contracture, the degree of collagen deposition, the number of total cells, the MOD of RAS and ERK2, and the expression levels of TGF-β1, FGF-2, and ERK2 were analyzed by ANOVA analysis across the groups. The difference was statistically significant with *P* value of less than 0.05 (*P* < 0.05).

## Results

### Total contracture and arthrogenic contracture

Degrees of total and arthrogenic contracture in each group are illustrated in Table 2. Total and arthrogenic contracture in the IM and NR groups were significantly greater than in the C group (*P* < 0.05). Compared with the IM group, total contracture in the NR group was improved (*P* < 0.05), but there was no statistical significance when comparing arthrogenic contracture (*P* > 0.05). Compared with the NR group, total and arthrogenic contracture of the three intervention groups were improved (*P* < 0.05), they were significantly smaller in the CI group than in the EI group (*P* < 0.05) and the SI group (*P* < 0.05). Compared with the EI group, total contracture in the SI group was improved (*P* < 0.05), but there was no statistical significance when comparing arthrogenic contracture (*P* > 0.05).

**Table 2:** Results of the total contracture and arthrogenic contracture

		**Degree of contracture (∘)**	
**Grouping**	**Quantity**	**Total contracture**	**Arthrogenic contracture**
Group C	6	0 ± 0	0 ± 0
Group IM	6	78.89 ± 3.35^a^	44.08 ± 2.73^a^
Group NR	6	70.42 ± 4.39^ab^	45.13 ± 3.49^a^
Group EI	6	61.87 ± 3.75^abc^	36.97 ± 2.51^abc^
Group SI	6	52.82 ± 4.65^abcd^	37.54 ± 3.14^abc^
Group CI	6	43.97 ± 5.27^abcde^	27.98 ± 3.18^abcde^

Data are expressed as mean ± standard deviation. ^a^*P* < 0.05 vs the C group, ^b^*P* < 0.05 vs the IM group, ^c^*P* < 0.05 vs the NR group, ^d^*P* < 0.05 vs the EI group, ^e^*P* < 0.05 vs the SI group. C: Control group; IM: Immobilization model group; NR: Natural recovery group; EI: Extracorporeal shock wave intervention group; SI: Static progressive stretching intervention group; CI: Static progressive stretching combined with extracorporeal shockwave intervention group.

**Figure 3.**
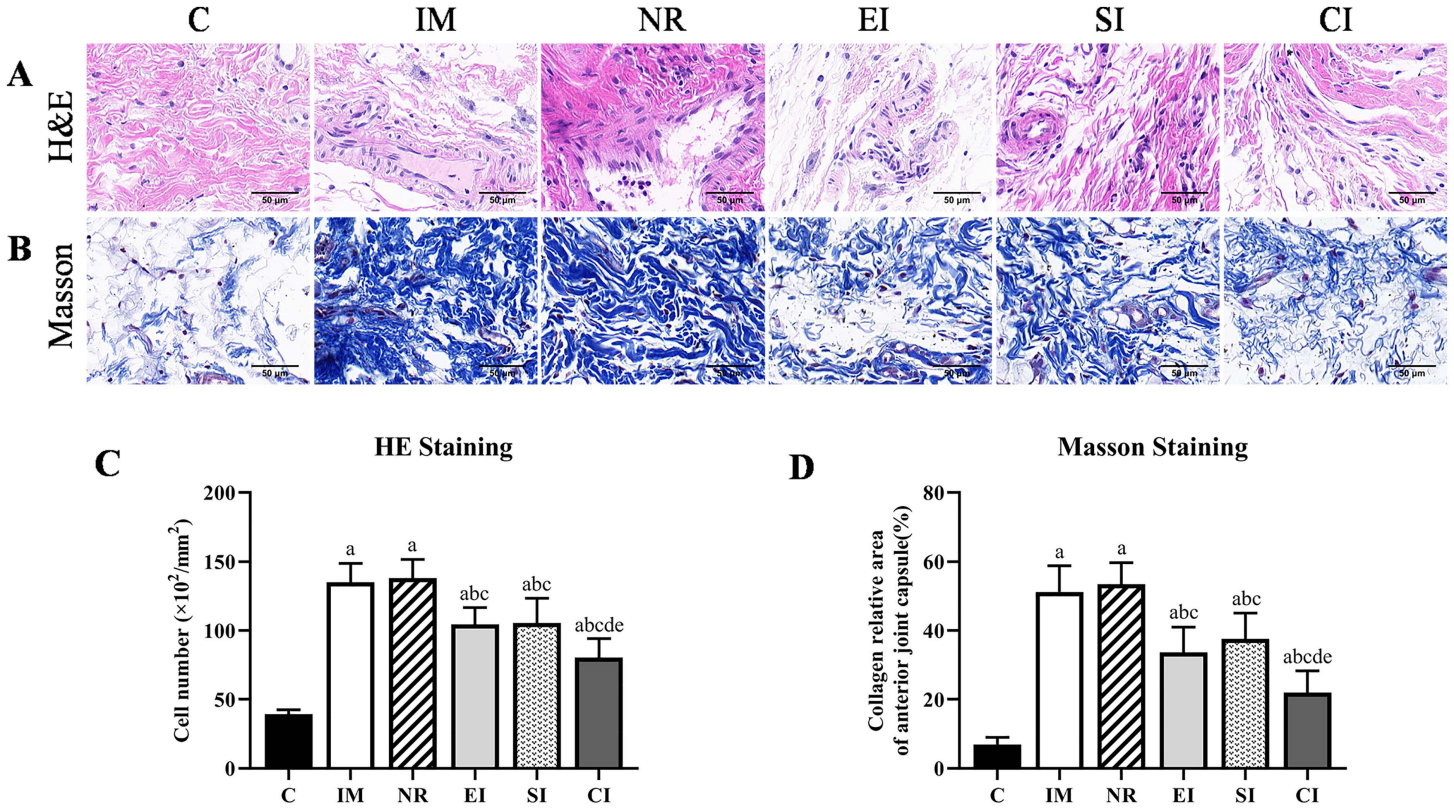
**H&E staining and Masson staining.** (A) Results of H&E staining; (B) Results of Masson staining; (C) The quantitative analysis of the number of total cells in each group; (D) The quantitative analysis of the percentage of collagen deposition (blue area) in each group. Data are expressed as mean ± standard deviation. ^a^*P* < 0.05 vs the C group, ^b^*P* < 0.05 vs the IM group, ^c^*P* < 0.05 vs the NR group, ^d^*P* < 0.05 vs the EI group, ^e^*P* < 0.05 vs the SI group. Scale bars ═ 50 µm. C: Control group; IM: Immobilization model group; NR: Natural recovery group; EI: Extracorporeal shock wave intervention group; SI: Static progressive stretching intervention group; CI: Static progressive stretching combined with extracorporeal shock wave intervention group.

### Cell count

The results of H&E staining and quantitative analysis are shown in Figure 3A and 3C. Compared with the C group, the number of total cells in the IM group and the NR group significantly increased (*P* < 0.05). Spindle-shaped fibroblast-like cells were observed in both IM and NR groups and increased significantly, while the inflammatory cell infiltration was observed in the NR group, but there was no statistical significance between the two groups (*P* > 0.05). Compared with the NR group, the number of total cells in the other three intervention groups was decreased (*P* < 0.05), and the number of total cells in the CI group was significantly smaller than in the EI group (*P* < 0.05) and the SI group (*P* < 0.05). There was no statistical significance in the number of total cells between the EI and the SI groups (*P* > 0.05).

### Collagen deposition

The results of Masson staining and quantitative analysis are shown in Figure 3B and 3D. Compared with the C group, the collagen deposition in the IM group and the NR group was obvious (*P* < 0.05), but there was no statistical significance between these two groups (*P* > 0.05). Compared with the NR group, the collagen deposition in the three intervention groups was decreased (*P* < 0.05), and the collagen deposition in the CI group was smaller than in the EI group (*P* < 0.05) and the SI group (*P* < 0.05). There was no statistical significance in the collagen deposition between the EI and the SI groups (*P* > 0.05).

### The mean optical density of RAS and ERK2

**Figure 4.**
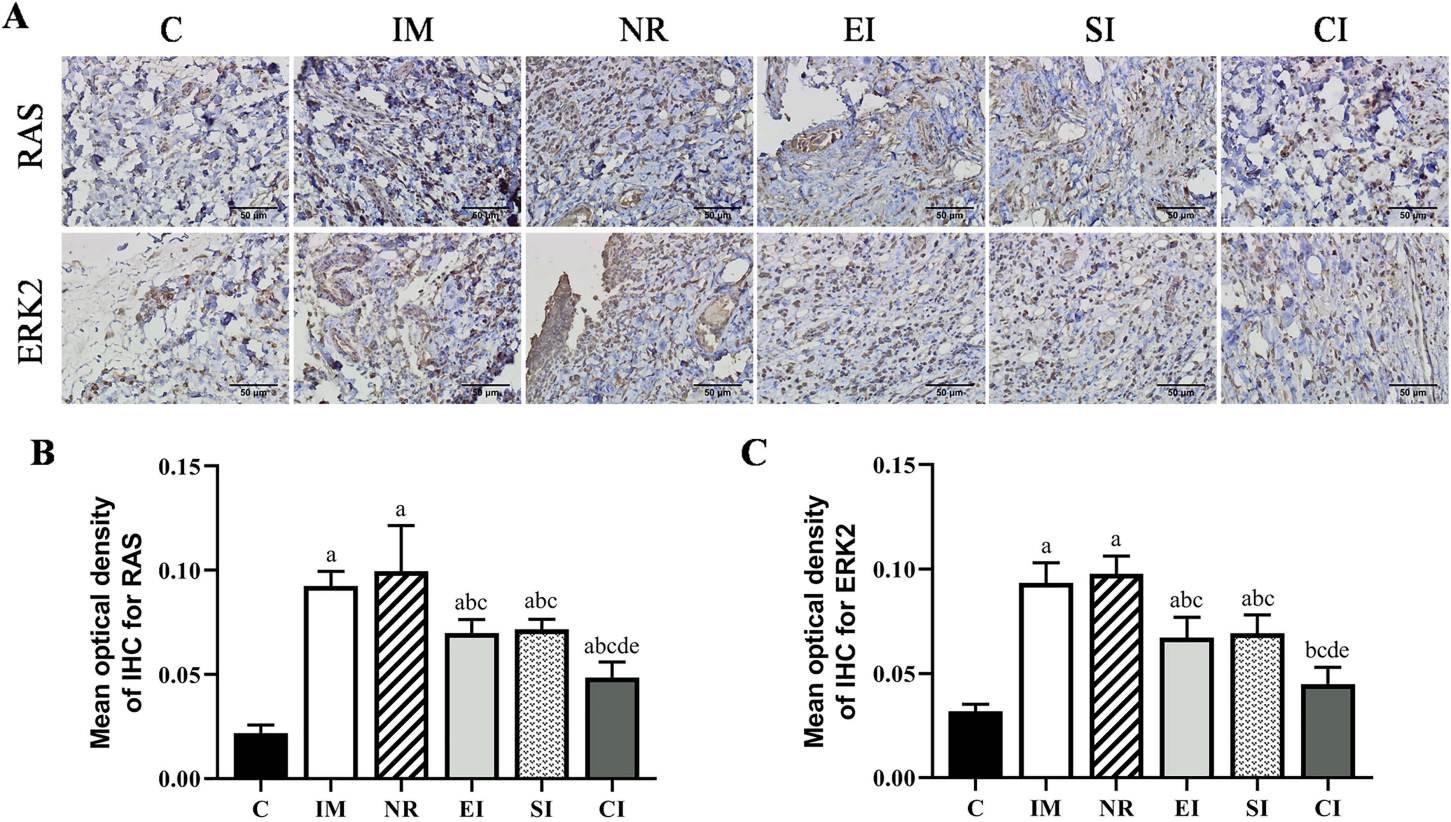
**Immunohistochemical analysis**. (A) Results of immunohistochemical staining of the upstream protein RAS and downstream protein ERK2 in the MAPK/ERK pathway. Scale bars ═ 50 µm; (B) The quantitative analysis of the mean optical density of RAS positive expression; (C) The quantitative analysis of the mean optical density of ERK2 positive expression. Data are expressed as mean ± standard deviation. ^a^*P* < 0.05 vs the C group, ^b^*P* < 0.05 vs the IM group, ^c^*P* < 0.05 vs the NR group, ^d^*P* < 0.05 vs the EI group, ^e^*P* < 0.05 vs the SI group. C: Control group; IM: Immobilization model group; NR: Natural recovery group; EI: Extracorporeal shock wave intervention group; SI: Static progressive stretching intervention group; CI: Static progressive stretching combined with extracorporeal shock wave intervention group; IHC: Immunohistochemistry; ERK2: Extracellular signal-regulated kinase 2.

To explore the role of MAPK/ERK pathway in the development and improvement of joint contracture, immunohistochemical staining was performed on the anterior joint capsule (Figure 4A). The analysis of the MOD of the upstream protein RAS in this signaling pathway is shown in Figure 4B. Compared with the C group, the MOD of RAS in the IM group and the NR group was significantly higher (*P* < 0.05), but there was no statistical significance between the two groups (*P* > 0.05). Compared with the NR group, the MOD of RAS in the other three intervention groups was reduced to varying degrees. It was lower in the CI group than in the EI group (*P* < 0.05) and the SI group (*P* < 0.05), but there was no significant difference between the EI and the SI group. The variation tendency of the MOD of the downstream protein ERK2 was generally consistent with that of RAS, but there was no significant difference between the CI and the C group (*P* > 0.05) (Figure 4C).

### Expression of the protein levels of TGF-β1, FGF-2, and ERK2

**Figure 5.**
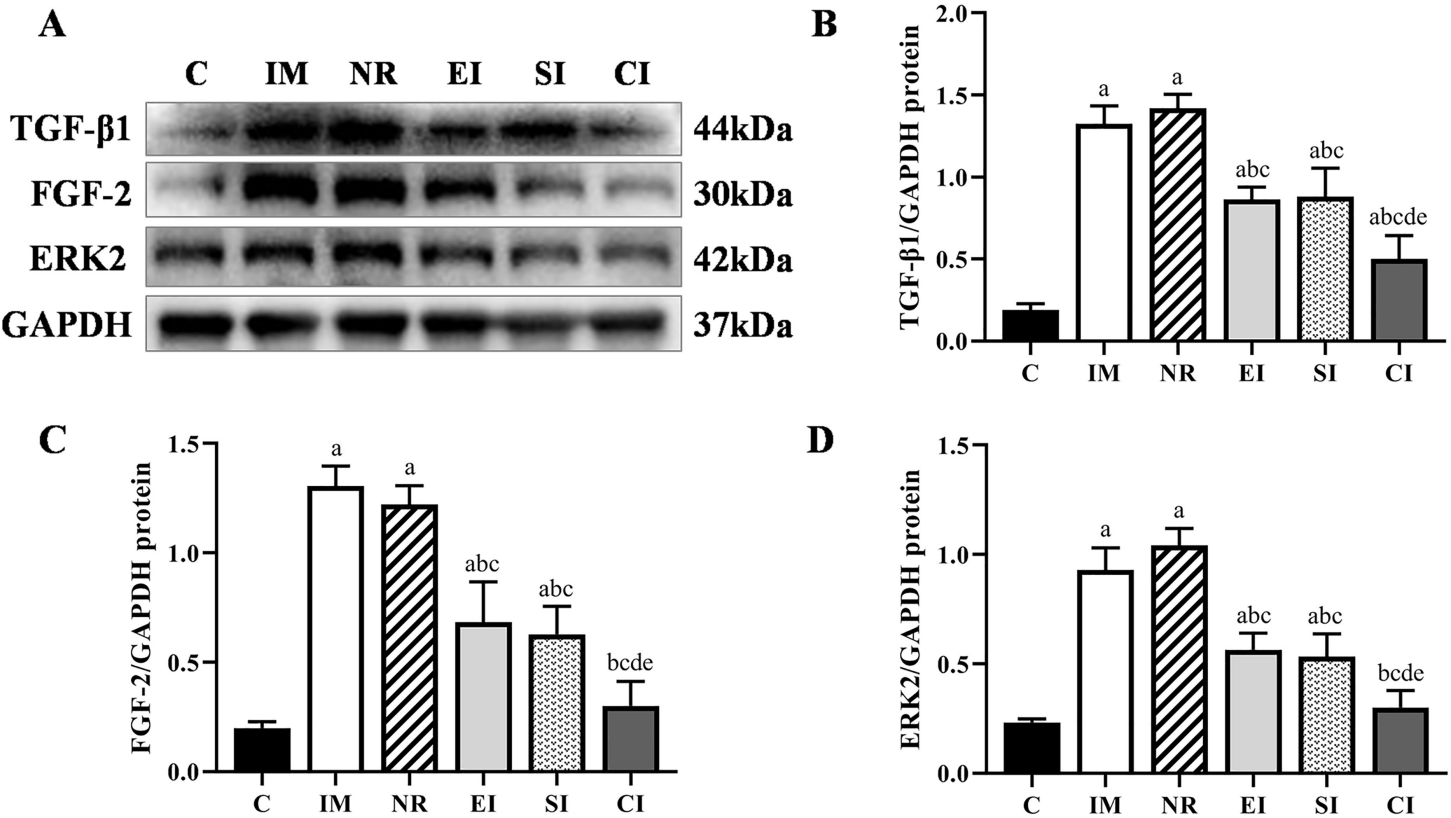
**Protein expression levels of TGF-β1, FGF-2, and ERK2 relative to GAPDH**. (A) Western blotting bands of TGF-β1, FGF2, ERK2, and GAPDH proteins; (B) The quantitative analysis of TGF-β1; (C) The quantitative analysis of FGF-2; (D) The quantitative analysis of ERK2. Data are expressed as mean ± standard deviation. ^a^*P* < 0.05 vs the C group, ^b^*P* < 0.05 vs the IM group, ^c^*P* < 0.05 vs the NR group, ^d^*P* < 0.05 vs the EI group, ^e^*P* < 0.05 vs the SI group. C: Control group; IM: Immobilization model group; NR: Natural recovery group; EI: Extracorporeal shock wave intervention group; SI: Static progressive stretching intervention group; CI: Static progressive stretching combined with extracorporeal shock wave intervention group; TGF-β1: Transforming growth factor beta-1; FGF2: Fibroblast growth factor 2; ERK2: Extracellular signal-regulated kinase 2; GAPDH: Glyceraldehyde-3-phosphate dehydrogenase.

The relationship between the average protein expression levels of TGF-β1, FGF-2, ERK2, and GAPDH in the six groups of specimens is shown in Figure 5A. Compared with the C group, the expression levels of TGF-β1 in the IM and the NR group were significantly increased (*P* < 0.05), but there was no difference between the two groups (*P* > 0.05). The expression levels of TGF-β1 in the three intervention groups were significantly lower than in the NR group, but none of the groups returned to the levels of the C group. The difference between the EI and the SI group was not statistically significant (*P* > 0.05). It was worth noting that the expression levels of TGF-β1 in the CI group were lower than in the EI group (*P* < 0.05) and the SI group (*P* < 0.05) (Figure 5B). The variation tendency of the protein expression levels of FGF-2 and ERK2 was generally consistent with that of TGF-β1, but there was no significant difference between the CI and the C group (*P* > 0.05) (Figure 5C and 5D).

## Discussion

Joint contracture is common complication in clinical practice, which plagues rehabilitation and orthopedics. Stretch therapy is the main method for preventing and treating joint contracture, and SPS is a good choice when the effect of routine treatment is at a plateau [24–26, 28]. ESWT is widely used in the treatment of musculoskeletal diseases as a safe non-invasive treatment, which generates intercellular and extracellular responses that can relieve pain, promote tissue regeneration, promote bone healing, and destroy calcium deposits in musculoskeletal structures [29]. It is also a safe and effective non-invasive treatment for many fibrotic diseases such as myogenic contracture, pathological scars, epidural fibrosis, etc. [13–20]. In this study, extension knee joint contracture in rats was treated in different ways, to explore whether the treatment effect of SPS combined with ESWT is better than single treatment, and to guide clinical practice.

In this study, total and arthrogenic contracture in the IM group were significantly increased compared with the C group, suggesting that the knee joint movement disorder of the rat was obvious. After different ways of treatment, total contracture of each group was relieved, but it did not return to the level of the C group, indicating that the joint contracture of each group was not completely reversed. When the NR group was compared with the IM group, total contracture was relieved but the arthrogenic contracture was not, indicating that the myogenic contracture could be partially recovered by natural movement, but the arthrogenic contracture could not, which was consistent with the previous research results [5, 6, 30]. Total contracture was relieved in the SI group compared with the EI group, but there was no significant change in the arthrogenic contracture, which may be due to the improvement in myogenic factors, such as the soft tissue around the knee joint and the quadriceps during the stretching process. Compared with the NR group, the CI group had the most significant improvement in total and arthrogenic contracture of all the intervention groups, indicating that the combined intervention was significantly better than the single intervention, suggesting that ESWT combined with SPS will be more beneficial to the recovery of ROM.

Inflammatory and proinflammatory signaling cascades play an important role in the formation of joint fibrosis. The inflammatory response reaches the peak in the early stage of remobilization [6]. The increase of collagen content is closely related to fibroblasts, and fibroblasts migrate and accumulate around the joint capsule during the process of joint contracture [2], both of which are important factors in the formation of joint contracture [6, 31, 32]. The main phenotypes of macrophages are M1 and M2. M1 type has proinflammatory effect, whereas M2 type has anti-inflammatory properties. Low-energy ESWT can stimulate the polarity shift of macrophages from M1 to M2, resulting in a good anti-inflammatory effect [29]. Low-energy ESWT could also be effective in suppressing hypertrophic scar formation by inhibiting fibroblast density and α-SMA expression [33]. Rinella et al. [34] found that shock wave treatment could reduce the expression of integrin alpha 11, inhibit the development of a myofibroblast phenotype, and downregulate the expression of the myofibroblast marker α-SMA and the extracellular matrix protein type I collagen. It is well known that stretching can directly promote the regression of local inflammation in connective tissues. Berrueta et al. [35] found that acute inflammation and neutrophil migration were reduced after stretching treatment of connective tissue. In this study, the number of total cells and collagen deposition were significantly increased in the IM group after four weeks of immobilization. The spindle-shaped fibroblast-like cells and inflammatory cell infiltration were obvious in the NR group after four weeks of natural recovery, and the number of total cells and collagen deposition did not change significantly compared with the IM group, suggesting that neither of them could be recovered through natural activity. The three intervention groups achieved effective remission after different treatments. When SPS was combined with ESWT, the number of total cells and collagen deposition reached the lowest level, which was consistent with the remission trend of arthrogenic contracture, suggesting that the combination of ESWT and SPS will more effectively reduce the total cells and collagen deposition in the joint capsule, and the reduction of both may be one of the reasons for the relief of arthrogenic contracture.

TGF-β1 can stimulate collagen expression in fibroblasts through the Smad pathway and cooperate with FGF-2 to stimulate fibroblast proliferation through the MAPK/ERK pathway. ERK can be divided into ERK1 and ERK2, among which ERK2 plays a leading role in the formation of joint capsule fibrosis and joint contracture [11]. Inhibition of ERK2 expression could control fibroblast proliferation and collagen production and effectively reduce the formation of joint adhesions and joint capsule fibrosis [11, 12]. Therefore, TGF-β1, FGF-2, and ERK2 play important roles in the occurrence and development of joint capsule fibrosis. In this study, the expression levels of target protein increased significantly after the development of joint contracture. Except for the NR group, the expression levels of target protein in the three intervention groups were inhibited effectively, and the CI group had the lowest expression levels, which was consistent with the variation tendency of collagen deposition and the number of total cells. It is suggested that ESWT combined with SPS could inhibit the expression of TGF-β1, FGF-2, and ERK2 more effectively, which may be one of the molecular mechanisms for the alleviation of collagen deposition and cell proliferation.

Hypoxic environment is induced during immobilization [13, 14], and inflammatory response is generated during remobilization [6], both of which are the main stimulators for the activation of the MAPK/ERK pathway. A large number of studies have demonstrated that the overactivated MAPK/ERK pathway is involved in the fibrosis process of different tissues and organs [8–10, 36, 37]. In this study, the MOD of the upstream protein RAS and the downstream protein ERK2 in the MAPK/ERK pathway was significantly increased after four weeks of the knee joint immobilization, which was consistent with the development trend of the arthrogenic contracture, the number of total cells, the collagen deposition, and the expression levels of each detected protein. It is suggested that the overactivated MAPK/ERK pathway may be one of the molecular mechanisms underlying the development of joint contracture, and there may be an intricate relationship between the overactivated MAPK/ERK pathway and the expression of each detected protein and histopathological changes. The MOD of RAS and ERK2 of three intervention groups was reduced effectively, and the CI group had the lowest MOD, suggesting that both ESWT and SPS could inhibit the overactivated MAPK/ERK pathway, and that the inhibition was more effective after combined treatment, which may be one of the reasons for the improvement of joint contracture.

There are several limitations in this study. Firstly, SPS is a manual intervention because of the limitation of experimental equipment. The drafting torque cannot be clearly defined. It is necessary to design corresponding experimental equipment to quantify it, which is the focus of the next work. Secondly, this study only explored the number of total cells and collagen expression of the joint capsule by H&E and Masson staining. Further research is needed to clarify the expression levels of related inflammatory factors, both proteins and mRNA levels, and the proliferation of related cells. Finally, in addition to the joint capsule, arthrogenic components also include bone, cartilage, synovium, and ligaments. Further research on them will help to understand the mechanism of arthrogenic contracture.

## Conclusion

This research suggests that overactivated MAPK/ERK pathway may be one of the molecular mechanisms in the formation of joint contracture. Both ESWT and SPS treatment can improve arthrogenic contracture in rats, which may inhibit the high expression of TGF-β1, FGF-2, and ERK2 in the joint capsule and overactivated MAPK/ERK pathway, and the therapeutic effect of the combined treatment was better than that of ESWT or SPS alone. We believe that ESWT combined with SPS is reasonable in the treatment of arthrogenic contracture induced by immobilization, and it is worth promoting in clinical treatment.
